# Supplementary material for: Extracting seizure onset from surface EEG with independent component analysis: Insights from simultaneous scalp and intracerebral EEG
Source: Neuroimage Clin. 2021 Sep 29;32:102838. doi: 10.1016/j.nicl.2021.102838 (PMC8503578; doi:10.1016/j.nicl.2021.102838)
Supplement: Supplementary data 1 [file mmc1.pdf]

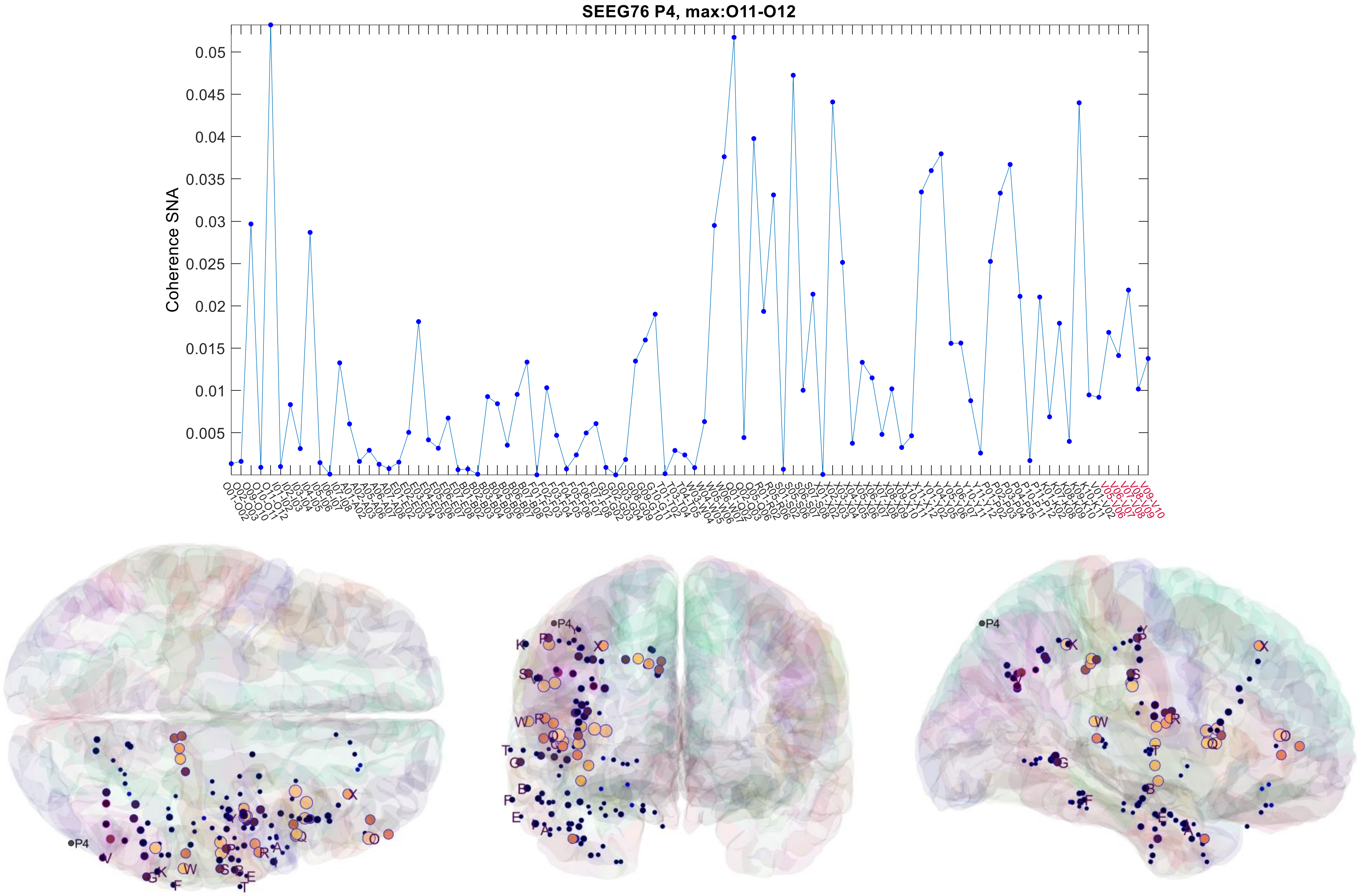

Supplementary Figure S1. Coherence of signal on scalp electrode P4 with intracranial electrodes in patient 3; A) top, profile of the coherence, quantified by the significant normalized area (SNA) in time-frequency plane; B-D)) bottom row, 3D representation of SEEG electrodes (blue - pink), in MNI space, in axial, coronal and sagittal views; the size and brightness of the electrode markers is proportional to the coherence with P4.

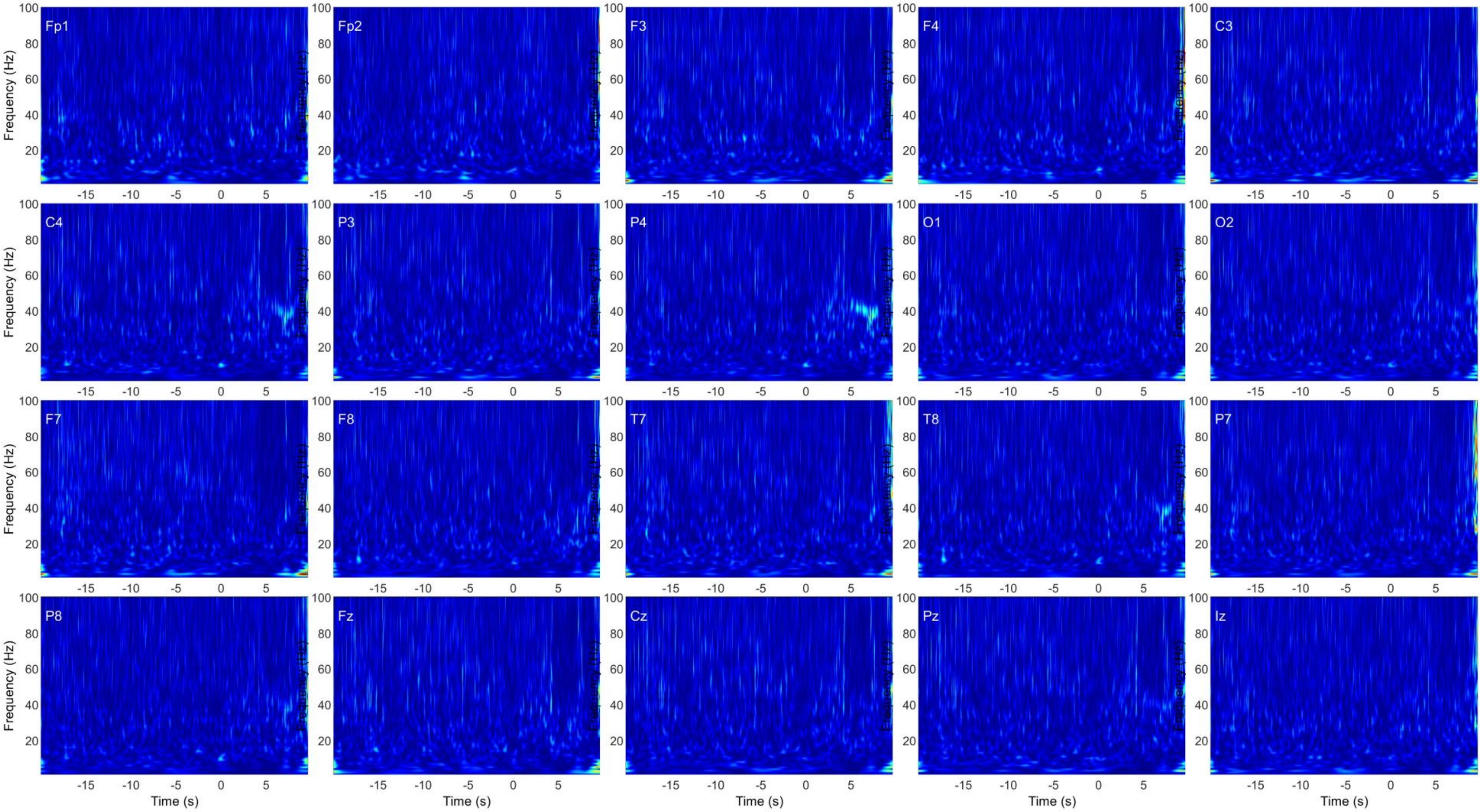

Supplementary Figure S2. Time-frequency maps (using Z-score normalization) of the scalp EEG recordings (first 20 out of 36) in patient 7 showing a ~40-Hz LVFA pattern visible on electrode P4.

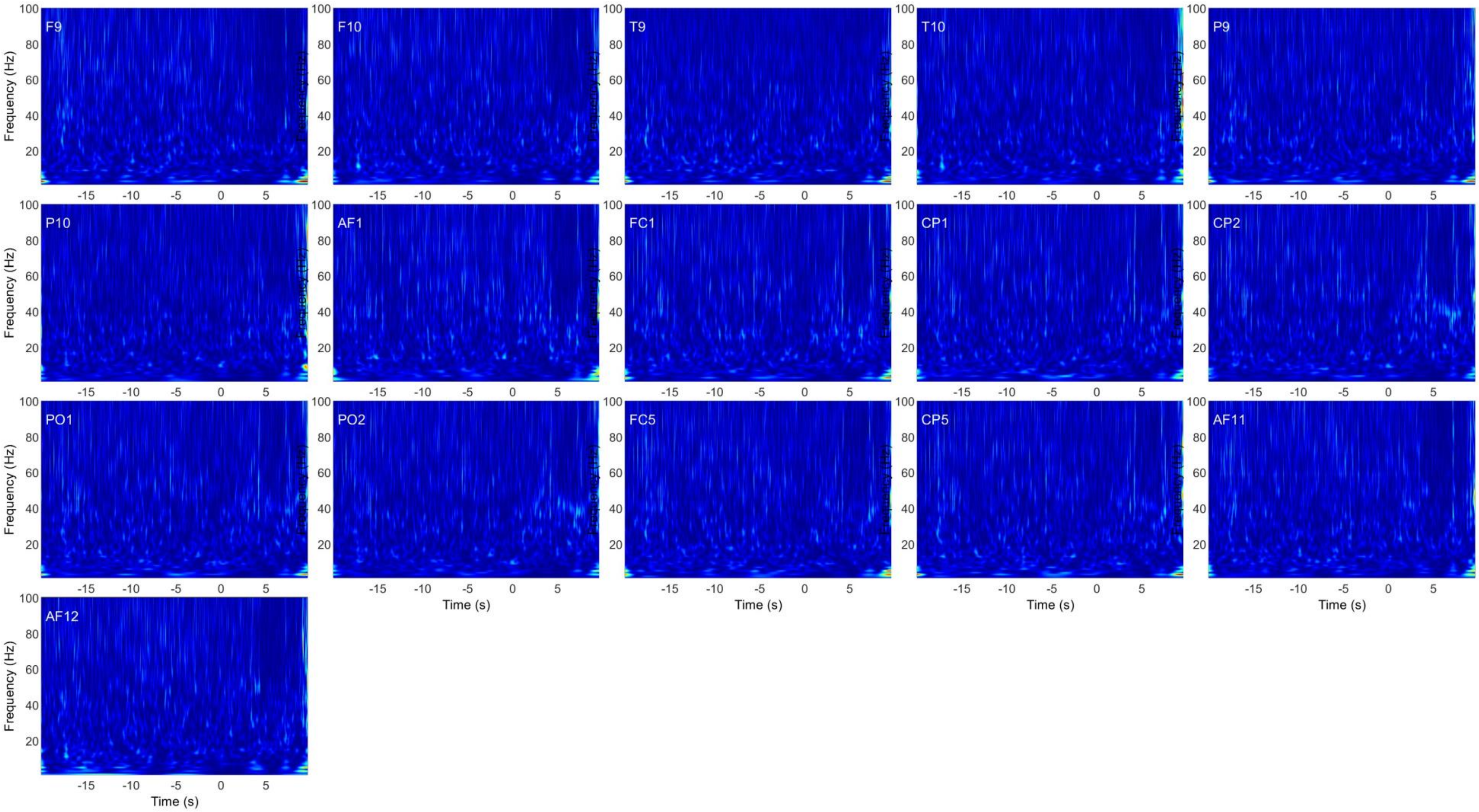

Supplementary Figure S3. Time-frequency maps (using Z-score normalization) of the scalp EEG recordings (channels 21 through 36) in patient 7, showing that LVFA patterns are not clearly visible on this signal set.

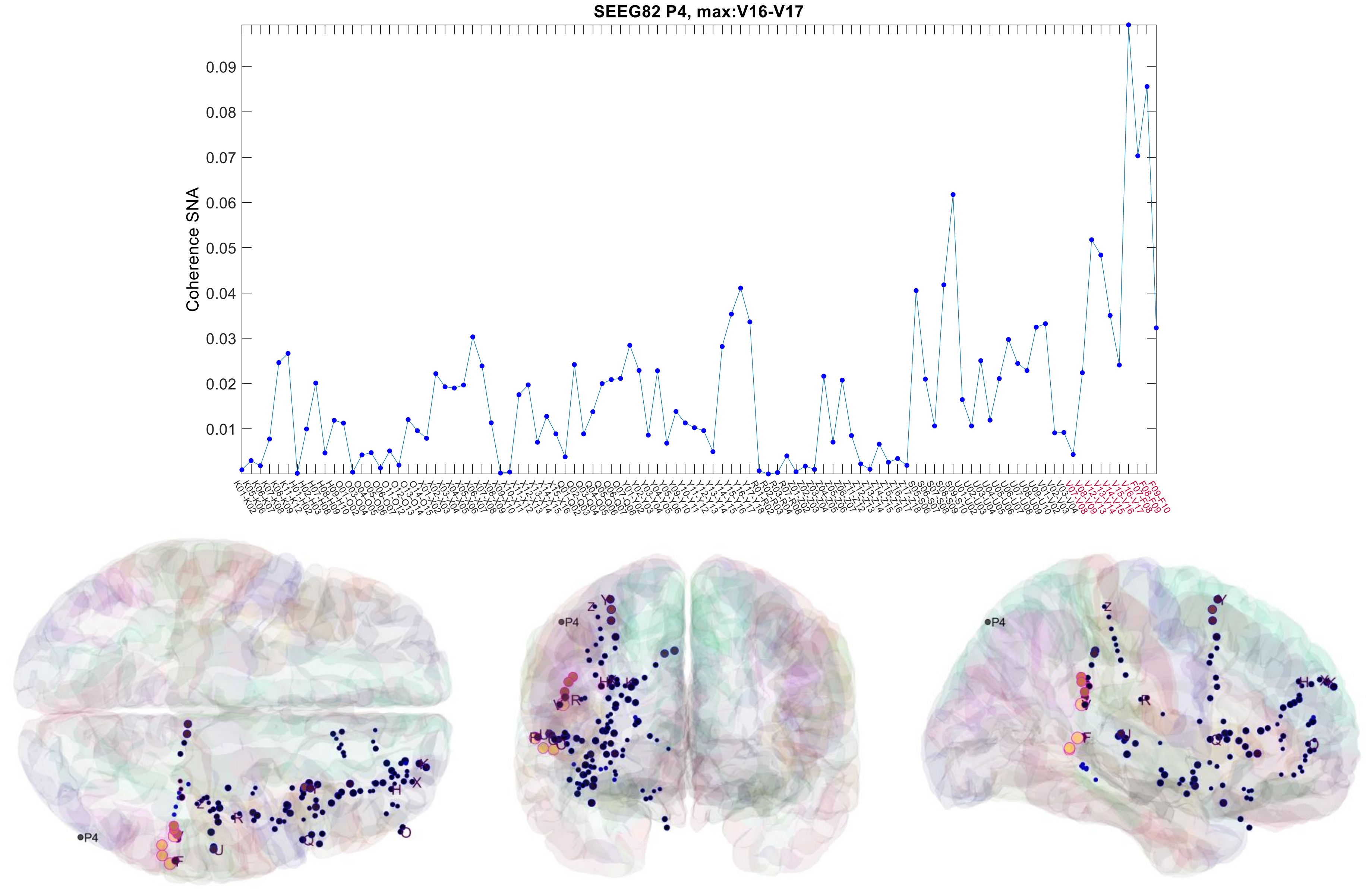

Supplementary Figure S4. Coherence of signal on scalp electrode P4 with intracranial electrodes in patient 7; A) top, profile of the coherence, quantified by the significant normalized area (SNA) in time-frequency plane; B-D)) bottom row, 3D representation of SEEG electrodes (blue - pink), in MNI space, in axial, coronal and sagittal views; the size and brightness of the electrode markers is proportional to the coherence with P4.

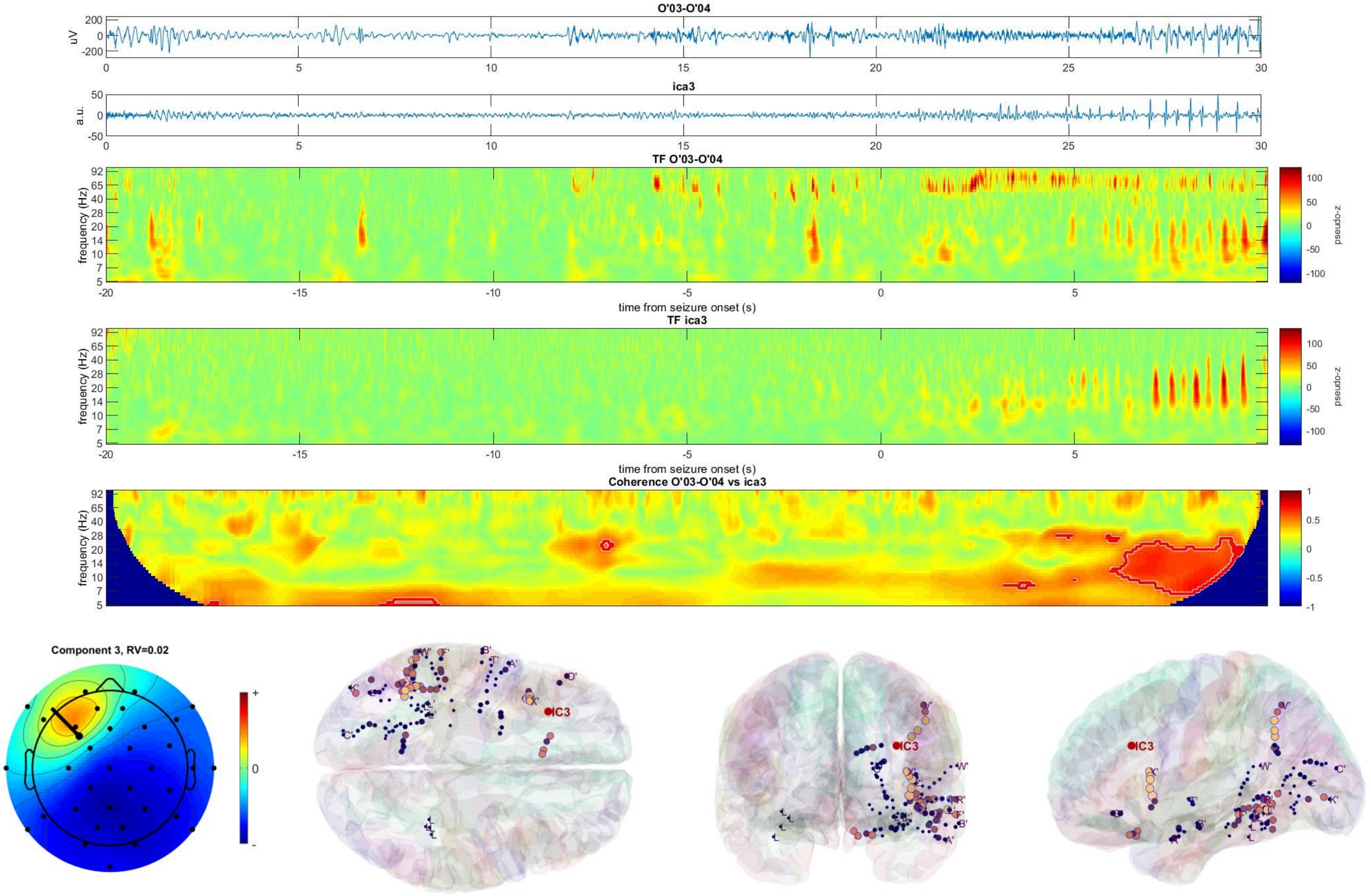

Supplementary Figure S5. Exemple of an ictal discharge in the SOZ of patient 12 (contacts O’03-O’04 located in orbitofrontal cortex) containing an initial LVFA followed by low-frequency repetitive discharges that propagate primarily to the insular cortex. Only low-frequency discharges originating from this deep source is visible on scalp and captured by the independent component 3. None of the other components capture LVFA.

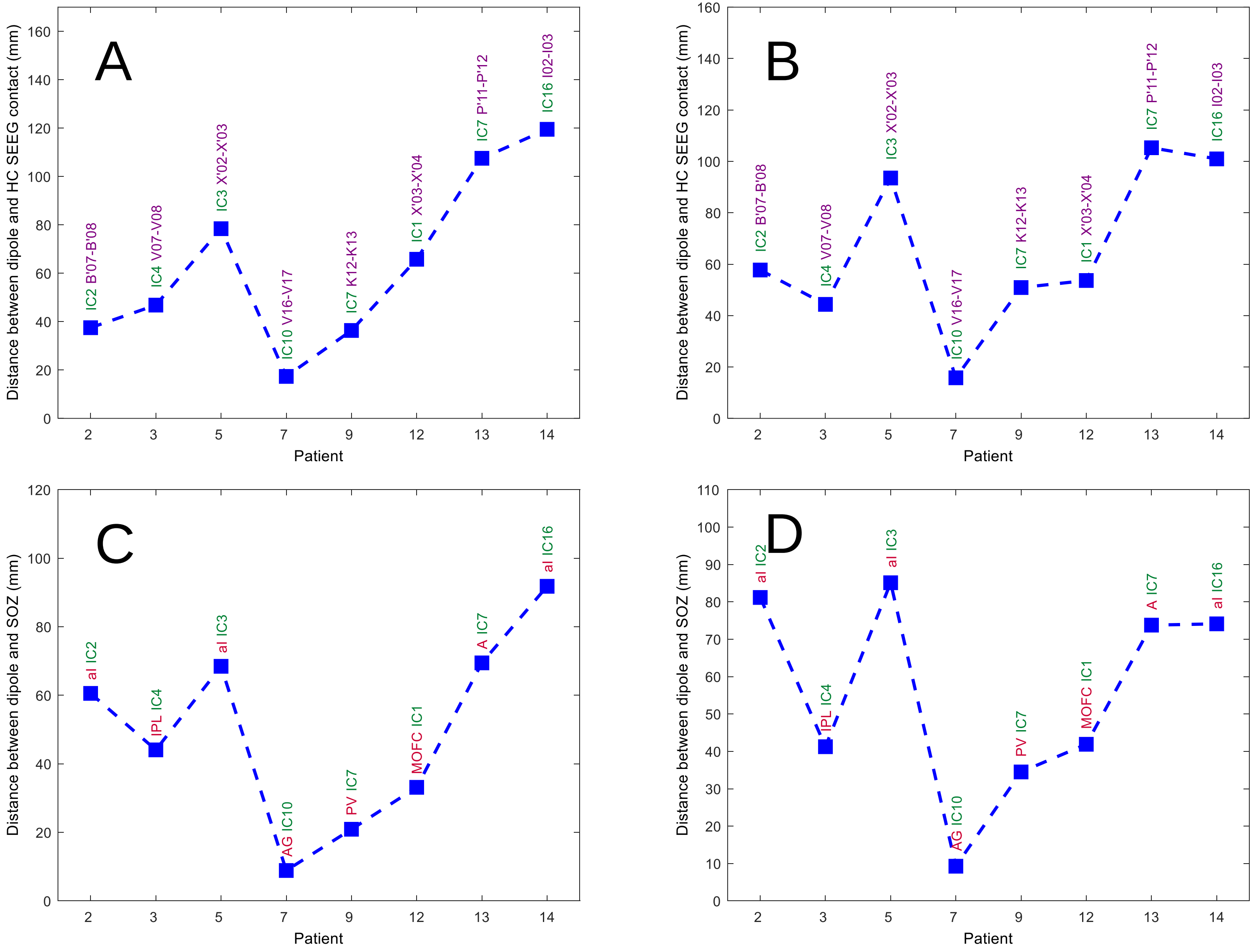

Supplementary Figure S6. Results of distributed source localization; A-B) the distance in MNI space between component's peak activation (A – sLoreta, B – beamformer LCMV) and SEEG pair exhibiting highest coherence (HC) with the component; C-D) distance between component's peak activation and SOZ location (C – sLoreta, D – beamformer LCMV). Abbreviations used: A – amygdala, AG – angular gyrus, aI – anterior insula, IPL – inferior parietal lobule, PV – periventricular nodular heterotopia (in patient 9), MOFC – medial orbito-frontal cortex.
